# Structural and functional characterization of CMP-*N*-acetylneuraminate synthetase from *Vibrio cholerae*

**DOI:** 10.1107/S2059798319006831

**Published:** 2019-05-31

**Authors:** Sucharita Bose, Debayan Purkait, Deepthi Joseph, Vinod Nayak, Ramaswamy Subramanian

**Affiliations:** a Institute for Stem Cell Science and Regenerative Medicine, GKVK Post, Bellary Road, Bangalore 560 065, India

**Keywords:** CMP-*N*-acetylneuraminate synthetase, *Vibrio cholerae*, sialic acid, structure, function

## Abstract

Several pathogenic bacteria utilize sialic acid, including host-derived *N*-acetylneuraminic acid (Neu5Ac), in at least two ways: they use it as a nutrient source and as a host-evasion strategy by coating themselves with Neu5Ac. Given the significant role of sialic acid in pathogenesis and host-gut colonization by various pathogenic bacteria, including *Neisseria meningitidis*, *Haemophilus influenzae*, *Pasteurella multocida* and *Vibrio cholerae*, several enzymes of the sialic acid catabolic, biosynthetic and incorporation pathways are considered to be potential drug targets. In this work, findings on the structural and functional characterization of CMP-*N*-acetylneuraminate synthetase (CMAS), a key enzyme in the incorporation pathway, from *Vibrio cholerae* are reported. CMAS catalyzes the synthesis of CMP-sialic acid by utilizing CTP and sialic acid. Crystal structures of the apo and the CDP-bound forms of the enzyme were determined, which allowed the identification of the metal cofactor Mg^2+^ in the active site interacting with CDP and the invariant Asp215 residue. While open and closed structural forms of the enzyme from eukaryotic and other bacterial species have already been characterized, a partially closed structure of *V. cholerae* CMAS (VcCMAS) observed upon CDP binding, representing an intermediate state, is reported here. The kinetic data suggest that VcCMAS is capable of activating the two most common sialic acid derivatives, Neu5Ac and Neu5Gc. Amino-acid sequence and structural comparison of the active site of VcCMAS with those of eukaryotic and other bacterial counterparts reveal a diverse hydrophobic pocket that interacts with the C5 substituents of sialic acid. Analyses of the thermodynamic signatures obtained from the binding of the nucleotide (CTP) and the product (CMP-sialic acid) to VcCMAS provide fundamental information on the energetics of the binding process.

## Abstract 2

CMP-*N*-acetylneuraminate synthetase (CMAS) is a key enzyme in the sialic acid incorporation pathway and plays a crucial role in the virulence and survival of several pathogenic bacteria. Here, the structural and functional properties of CMAS from the pathogenic bacterium *Vibrio cholerae* are reported. Upon CDP binding, a partial domain closure is observed that was previously unreported in homologous structures. Kinetic studies reveal that the enzyme shows substrate promiscuity and can activate both Neu5Ac and Neu5Gc.

## 1. Introduction   

Sialic acids are a family of nine-carbon α-keto sugar acids that are present at the terminal positions of various glycoconjugates on the eukaryotic cell surface and are functionally significant in several physiological and pathological processes (Varki, 2008 ▸). *N*-Acetylneuraminic acid (Neu5Ac) is the most abundant form of sialic acid. The bacterial pathogens that colonize the heavily sialylated niche of the mammalian gut and respiratory tract have co-evolved to scavenge sialic acid from the eukaryotic host and use it as a carbon and nitrogen source (Severi *et al.*, 2007 ▸). Several opportunistic bacteria including *Neisseria meningitidis*, *Escherichia coli* K1, *Haemophilus influenzae*, *Haemophilus ducreyi* and *Pasteurella multocida* ‘sugar-coat’ themselves with host-derived Neu5Ac on various glycoconjugates on their surface to evade the host innate immune system (McDonald *et al.*, 2016 ▸). Sialylated glycoconjugates protect *Neisseria gonorrhoae* from phagocytosis (Rest & Frangipane, 1992 ▸). A study comparing the virulence of *Campylobacter jejuni* showed that the sialylated form (as compared with the nonsialylated form) of the organism is capable of invading human epithelial cell lines more effectively (Louwen *et al.*, 2008 ▸). These studies suggested that the enzymes of the Neu5Ac incorporation pathway play a critical role in bacterial virulence, pathogenesis and colonization, and hold promise as potential drug targets. The work reported here is part of our ongoing investigations into the structure–activity relationship of the enzymes involved in the uptake and incorporation of sialic acid into lipooligosaccharides (LOS) and lipopolysaccharides (LPS) from several pathogenic bacteria including *H. influenzae*, *P. multocida*, *Fusobacterium nucleatum* and *Vibrio cholerae*.

Cholera is a deadly enteric disease caused by *V. cholerae*, a Gram-negative bacterium that colonizes the human gut. Several studies have suggested that *V. cholerae* utilizes the sialic acid catabolic pathway to gain a competitive advantage over other pathogenic bacteria in host-gut colonization (Almagro-Moreno & Boyd, 2009 ▸). *Vibrio* pathogenicity island 2 (VPI-2), which is found exclusively among pathogenic strains of *V. cholerae*, contains a cluster of genes involved in the scavenging (*nanH*), transport (*dctPQM*) and catabolism (*nanA*, *nanE*, *nanK*, *nagA* and *nagB*) of sialic acid (Jermyn & Boyd, 2002 ▸; Almagro-Moreno & Boyd, 2009 ▸). A recent report showed that several members of the Vibrionaceae family possess a biosynthetic gene (*nab*) cluster and that the LPS of *Vibrio vulnificus* is decorated with sialic acid (Lewis *et al.*, 2011 ▸). In a mouse model of septicaemia, competition experiments revealed that the *V. vulnificus* sialic acid synthase mutant strain has a 300-fold lower chance of survival compared with the wild-type strain. Sialic acid plays a critical role in biofilm formation and motility, and also protects *V. vulnificus* from the host immune response (Lubin *et al.*, 2015 ▸). These data imply a critical role of the enzymes responsible for sialic acid utilization in the survival of *V. vulnificus*. A study from our laboratory has shown that *V. cholerae* can incorporate Neu5Ac into its LOS (Setty *et al.*, 2014 ▸). However, the enzymes of the sialic acid biosynthetic and incorporation pathway have not been explored in detail in *V. cholerae*.

**Figure 1:**
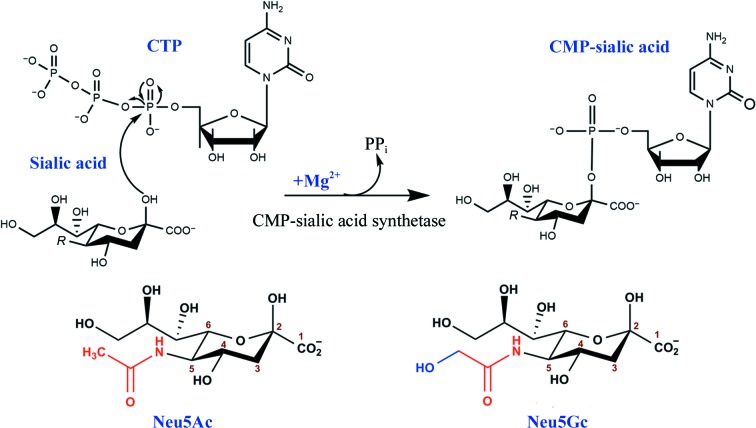
Schematic representation of the reaction catalyzed by CMP-sialic acid synthetase (CMAS). In the presence of Mg^2+^, CMAS catalyzes nucleophilic attack by the anomeric O atom of β-Neu5Ac on the α-phosphate of CTP, forming CMP-sialic acid and pyrophosphate (PP_i_). The natural sialic acid derivatives Neu5Ac and Neu5Gc with various substitutions at the C5 position (*R*) are shown. This figure is adapted from Sellmeier *et al.* (2015 ▸).

The first step of the incorporation pathway involves the activation of sialic acid to a cytidine monophosphate diester form catalyzed by CMP-sialic acid synthetase (EC 2.7.7.43), a ubiquitous enzyme that is found in both prokaryotes and eukaryotes (Sellmeier *et al.*, 2015 ▸; Mizanur & Pohl, 2008 ▸). In the presence of Mg^2+^, CMP-sialic acid synthetase (CMAS) catalyzes a sequential ordered reaction (Samuels *et al.*, 1999 ▸) that involves a nucleophilic attack on the α-phosphate of CTP by the anomeric O atom of β-Neu5Ac and the production of CMP-sialic acid and pyrophosphate (PP_i_; Fig. 1 ▸). The activated sialic acid is then incorporated into LOS or LPS by sialyltransferases (Li & Chen, 2012 ▸). Despite the commonality in overall three-dimensional structure between the prokaryotic and eukaryotic CMAS enzymes, differences in the active-site architecture and especially the sialic acid-binding pocket (Mosimann *et al.*, 2001 ▸; Krapp *et al.*, 2003 ▸) make the prokary­otic CMAS a viable drug target. CMAS is a unique enzyme as it recognizes a nonphosphorylated sugar molecule (sialic acid) and produces the monophosphorylated sugar instead of the diphosphorylated form that is found in nature (Kapitonov & Yu, 1999 ▸).

A *BLAST* search of the NCBI nonredundant database using the *P. multocida* CMAS protein sequence listed a similar protein that was annotated as a CMAS enzyme in *V. cholerae* (VcCMAS; NCBI WP_000064388.1). In this report, we present crystal structures of the apo form of the VcCMAS enzyme and of that complexed with cytidine diphos­phate (CDP) and Mg^2+^. We analyze the molecular basis of nucleotide and metal binding and compare our findings with the homologous *N. meningitidis* CMAS structure (NmCMAS; PDB entry 1eyr; Mosimann *et al.*, 2001 ▸). We also report the conformational changes in the dimerization domain of VcCMAS and the partial closure of the active site that are observed upon CDP binding. Such domain movement has not been reported in the homologous NmCMAS structures (PDB entries 1eyr and 6ckk; Mosimann *et al.*, 2001 ▸) and represents an intermediate state. We have also characterized the kinetic and thermodynamic properties of ligand binding to the VcCMAS enzyme and the results are presented here.

## 2. Materials and methods   

### 2.1. Expression and purification of VcCMAS in *Escherichia coli*   

The *V. cholerae* CMAS gene was synthesized and cloned into pET-300N DEST vector (Bairy *et al.*, 2018 ▸). Recombinant VcCMAS (Supplementary Fig. S2*a*) was overexpressed in *E. coli* Rosetta 2 DE3 cells grown in Luria–Bertani broth supplemented with 100 µg ml^−1^ ampicillin sodium salt and 30 µg ml^−1^ chloramphenicol. The culture was grown at 37°C until the OD reached 0.6–0.8, and protein expression was then induced with 0.5 m*M* IPTG for 16 h at 20°C. The cells were pelleted and resuspended in lysis buffer consisting of 70 m*M* Tris–HCl pH 8.0, 500 m*M* NaCl (buffer *A*) and lysed at 103 MPa (three passes) using an Emulsiflex C3 homogenizer (Avestin). The unlysed cells and cell debris were removed by centrifugation at 19 784*g* for 1 h. The supernatant was loaded onto a Ni–NTA column and washed with buffer *A* plus 20 m*M* imidazole, buffer *A* plus 50 m*M* imidazole and buffer *A* plus 100 m*M* imidazole. The protein was eluted in lysis buffer containing 250 m*M* imidazole. The fractions containing the protein were pooled, concentrated and injected onto a Superdex S200 16/60 size-exclusion (SEC) preparative column (GE Healthcare Life Sciences). The SEC buffer consisted of 50 m*M* Tris pH 8.0, 50 m*M* NaCl. The absorbance at 280 nm was used to determine the protein concentration following the Beer–Lambert relationship. The molar extinction coefficient was obtained using *ProtParam* on the ExPASy web server (Gasteiger *et al.*, 2005 ▸). One litre of *E. coli* culture yielded 10 mg of purified VcCMAS enzyme.

### 2.2. Kinetic assays   

The CMP-sialic acid synthetase enzymatic activity was determined from the initial rates of PP_i_ formation as detected using the EnzCheck pyrophosphatase assay kit from Invitrogen (Webb, 1992 ▸) using the manufacturer’s instructions with modifications. The EnzCheck reaction mixture was prepared in duplicate as a 200 µl reaction mixture consisting of 50 m*M* Tris–HCl pH 7.5, 1 m*M* MgCl_2_, 0.4 m*M* MESG substrate, 0.4 U purine nucleoside phosphorylase, 0.03 U inorganic pyrophosphatase and varying amounts of Neu5Ac or Neu5Gc and CTP and was incubated at 25°C for 10 min. The reaction was initiated by adding 100 ng of VcCMAS enzyme. The reaction was carried out at 25°C and the initial rate was calculated over a range of substrate concentrations (0–500 µ*M* for CTP and Neu5Ac; 0–3 m*M* for Neu5Gc). The concentration of the product 2-amino-6-mercapto-7-methylpurine formed by the enzymatic conversion of the substrate MESG was detected with a SpectraMax (Molecular Devices) at 360 nm. PP_i_ concentrations were calculated with a calibration curve plotted using PP_i_ standards. *V*_max_, *K*_m_ and *k*_cat_ values were calculated from two sets of data and were fitted to the Michaelis–Menten equation by nonlinear regression using *GraphPad Prism* 7.0 (GraphPad Software, La Jolla, California USA).

### 2.3. Isothermal titration calorimetry   

Isothermal titration calorimetry experiments were performed using a MicroCal ITC200 (GE Healthcare) at 25°C. In order to determine the respective binding partners, VcCMAS (50 µ*M*) was titrated independently against the reactant CTP (750 µ*M*) and the product CMP-sialic acid (700 µ*M*). Titration experiments with CTP were carried out in the presence of 5 m*M* MgCl_2_. For Neu5Ac titration experiments, the enzyme (100 µ*M*) was incubated with a threefold molar excess of CTP for 10 min and titrated against 1 m*M* Neu5Ac. The heat of dilution of the ligand was calculated from a control experiment and was subtracted from the data before curve fitting. The ITC data were fitted in *Origin* 7.0 (MicroCal) using the one-site binding equation. *NITPIC* was used for peak integration, and global weighted least-squares fitting of the thermograms was achieved with *SEDPHAT* (Zhao *et al.*, 2015 ▸). The reported affinity (*K*_a_), Δ*H* and Δ*S* values are an average from three independent experiments (*n* = 3) and the statistical error values were determined by Monte Carlo estimation (Bevington & Robinson, 2002 ▸).

### 2.4. Crystallization   

Hanging-drop vapor-diffusion experiments were performed using a Mosquito robot (TTP Labtech). Crystals of apo VcCMAS were obtained by mixing 0.5 µl screening solution with 0.5 µl VcCMAS protein solution (10 mg ml^−1^) and equilibrating the mixture against 100 µl of commercially available crystallization screen conditions. Rod-shaped crystals appeared within 2–3 days and grew to 0.3 × 0.05 × 0.05 mm in size in the presence of 200 m*M* calcium acetate, 0.1 *M* imidazole pH 8.0, 10% PEG 8000. Diffraction data were collected to 2.5 Å resolution from a single crystal of apo VcCMAS on the PROXIMA-1 beamline at the SOLEIL synchrotron source. The data showed anisotropic diffraction and were submitted to the *STARANISO* server for anisotropy correction (Tickle *et al.*, 2016 ▸).

Cocrystals were obtained by incubating VcCMAS (10 mg ml^−1^) with a 20-fold molar excess of the substrate analog cytidine diphosphate (CDP), Neu5Ac and Mg^2+^ for 1 h, and setting up hanging-drop vapor-diffusion trays as described above. Rod-shaped crystals appeared in 3–5 days in 0.1 *M* sodium cacodylate pH 6.5, 0.1 *M* calcium acetate, 10% PEG 8000 and grew to a maximum size of 0.1 × 0.1 × 0.05 mm. Prior to data collection, both the apo crystals and cocrystals were cryoprotected with 30% PEG 400 and flash-cooled in liquid nitrogen. Diffraction data were collected to 2.3 Å resolution from a single CDP-bound cocrystal on the ID29 beamline at the European Synchrotron Radiation Facility (ESRF). Both data sets were processed using *XDS*, *autoPROC* (Vonrhein *et al.*, 2011 ▸) and *AIMLESS* (Evans & Murshudov, 2013 ▸) in *CCP*4 (Winn *et al.*, 2011 ▸). Data-collection and processing statistics are summarized in Table 1 ▸. Despite being added to the protein solution, Neu5Ac was not observed in the crystal structure.

### 2.5. Structure solution   

The structure of apo VcCMAS was solved by molecular replacement with *Phaser* (McCoy *et al.*, 2007 ▸) in *PHENIX* (Adams *et al.*, 2010 ▸). The apo NmCMAS structure (PDB entry 1ezi; Mosimann *et al.*, 2001 ▸), which shares 40% amino-acid sequence identity with VcCMAS, was used as the search model. The initial model was built in *Coot* (Emsley & Cowtan, 2004 ▸); solvent molecules were added and refined using *phenix.refine* (Terwilliger *et al.*, 2008 ▸) and *autoBUSTER* (Smart *et al.*, 2012 ▸). The CDP-bound VcCMAS structure was determined by molecular replacement with *Phaser* using the refined model of apo VcCMAS as the search model. The initial difference density (*F*_o_ − *F*_c_ at 3.0σ) suggested that the residues (136–176) in the dimerization domain had undergone considerable motion, and they were subsequently built manually in *Coot*. Also, well defined positive *F*_o_ − *F*_c_ density (at 3.0σ) indicative of both bound CDP (subunits *A*, *B* and *C*) and Mg^2+^ (subunit *C*) was readily identified. To rule out other metals such as Mn^2+^ or Ca^2+^ and waters, we modeled each of these in the active site and refined them against the data set. With both Mn^2+^ and Ca^2+^ the *B* factor was much higher (106 and 88 Å^2^, respectively) than the average *B* factor of the surrounding residues. Modeling a water molecule at this position led to a *B* factor (56 Å^2^) that was lower than those of the coordinating residues and a residual positive *F*_o_ − *F*_c_ electron density (at 3.0 σ) that suggested the presence of a slightly heavier atom occupying this position. The ligands and the metal ion were built into the molecule using *Coot* and were refined against the 2.3 Å resolution data set. The refinement and Ramachandran statistics are outlined in Table 1 ▸. Models were validated using the wwPDB validation service. Atomic coordinates and structure factors were deposited in the Protein Data Bank as entries 6ifi and 6ifd for apo VcCMAS and the CDP–VcCMAS complex, respectively. All structural figures were prepared using the *PyMOL* molecular-graphics system (DeLano, 2002 ▸).

### 2.6. Molecular-docking studies   

The CMAS enzyme catalyzes a sequential ordered reaction in which the nucleotide binds prior to the sialic acid (Samuels *et al.*, 1999 ▸; Horsfall *et al.*, 2010 ▸). We used *AutoDock Vina* (Tanchuk *et al.*, 2015 ▸) to dock the naturally occurring sialic acid molecules Neu5Ac and Neu5Gc into the VcCMAS active site and *MGLtools* (Tanchuk *et al.*, 2015 ▸) and *PyMOL* (DeLano, 2002 ▸) to analyze the docking results. The CDP-bound VcCMAS was used as a template.

Previous studies show that the invariant Arg202 in the CMP-sialic acid-bound murine CMAS model (PDB entry 1qwj) moves more than 5 Å closer to the active site and the arginine residue hydrogen-bonds to the carboxylic acid group of Neu5Ac (Krapp *et al.*, 2003 ▸). The recently released structure of NmCMAS in complex with Neu5Ac and CMP also showed a similar movement of the catalytic arginine (Arg165; PDB entry 6ckl; M. M. Matthews, H., Yu, Y. Li, X. Chen & A. J. Fisher, unpublished work). To allow such flexibility, we divided the template into (i) a flexible part consisting of Arg169 (equivalent to Arg202 and Arg165) and Ser82 (equivalent to Ser81) and (ii) a rigid part consisting of the rest of the dimer.

## 3. Results and discussions   

### 3.1. Protein expression and purification   

A multiple sequence alignment (using *T-Coffee*; Notredame *et al.*, 2000 ▸) of the VcCMAS protein sequence with those of several prokaryotic and eukaryotic CMAS enzymes confirmed the presence of all five conserved motifs responsible for nucleotide and substrate binding in VcCMAS (Supplementary Fig. S1). The VcCMAS enzyme (Supplementary Fig. S2*a*) was expressed in *E. coli* Rosetta 2 DE3 cells and purified to single-band purity (Supplementary Fig. S2*b*) using affinity chromatography and size-exclusion chromatography. The protein eluted as a dimer from a preparative SEC column (Supplementary Fig. S2*c*).

### 3.2. Overall fold   

**Figure 2:**
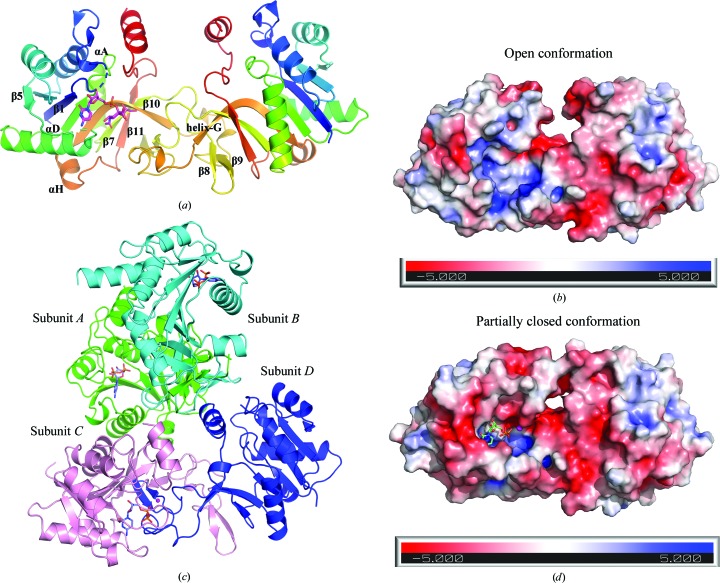
Overall structure of the VcCMAS enzyme. (*a*) Cartoon representation of the apo VcCMAS dimer. Each subunit is colored as a rainbow. CMP-sialic acid is modeled in the active site. The helices and β-strands mentioned in the text are labeled. (*b*) The electrostatic surface potential map of the apo structure highlights the open conformation of the enzyme. The negatively charged regions are colored red, neutral regions white and positively charged regions blue. (*c*) Cartoon representation of the CDP-bound VcCMAS tetramer observed in the asymmetric unit. The four subunits are colored blue, pink, green and cyan and are labeled *A*, *B*, *C* and *D*. The CDP present in the active site is shown in stick representation. (*d*) The electrostatic surface potential map of the CDP-bound structure shows the ‘partially closed’ conformation of the enzyme.

The tetragonal crystals (space group *P*4_1_) of the apoprotein contain a dimeric species in the asymmetric unit (Fig. 2 ▸ *a*). The two monomers bear a close structural resemblance to each other and superpose with a low r.m.s. deviation of 0.64 Å over 210 C^α^ atoms. The final refined apo VcCMAS model contains protein residues 3–226 (subunit *A*) and 3–228 (subunit *B*) plus two Ca^2+^ ions. Residues 13–20 in the phosphate-binding (P) loop and residues 74–79 are disordered in the apo structure.

The orthorhombic crystals (space group *P*2_1_2_1_2_1_) of the CDP-bound enzyme contain four molecules in the asymmetric unit arranged as dimer of dimers (*AB* and *CD*). The dimer–dimer association involves a shallow interface of 831 Å^2^ (Fig. 2 ▸ *c*). The tetrameric form is possibly a result of crystal-packing interactions as this oligomerization state does not correlate with the SEC result, which suggests that VcCMAS exists as a dimer in solution (Supplementary Fig. S2*c*). The final refined CDP-bound VcCMAS model contains protein residues 3–228 in subunits *A* and *D*, 0–233 in subunit *B* and 1–227 in subunit *C* plus three CDP molecules, one Mg^2+^ ion, one Ca^2+^ ion and one tetraethylene glycol (PG4), one triethylene glycol (PGE) and two ethylene glycol (EDO) molecules. The P-loop residues 13–20 are disordered in subunit *D*, which lacks CDP in the active site. Residues 164–172 in the dimerization domain of subunit *A* are also disordered.

The overall dimensions of the VcCMAS dimer are 86 × 40.6 × 34.2 Å. Each monomer is composed of a 196-residue compact globular domain (residues 1–136 and 177–234) and a 39-residue extended dimerization domain (residues 137–176). VcCMAS shares considerable overall structural similarity with murine CMAS and NmCMAS (C^α^ r.m.s. deviations of 1.6 and 1.4 Å, respectively). Similar to previously published CMAS structures (Krapp *et al.*, 2003 ▸; Mosimann *et al.*, 2001 ▸), the globular domain is structurally characterized as a three-layered α–β–α sandwich domain with a central β-sheet comprising six parallel β-strands and one antiparallel β-strand (β10) sandwiched between α-helices on both sides (Fig. 2 ▸ *a*). The secondary-structural elements are numbered following the same numbering scheme as used previously (Mosimann *et al.*, 2001 ▸). The dimerization domain, comprising of a 3_10_-helix (helix G), two antiparallel β-strands (β8 and β9) and associated loops, extends away from the central β-sheet in a domain-swapped fashion and interacts with the globular domain of the other protomer. Several main-chain antiparallel hydrogen-bond interactions between β7 and β10 along with side-chain hydrogen-bond interactions, salt bridges and hydrophobic interactions stabilize the dimerization domain and bury ∼1429 Å^2^ of each monomer in the dimer interface. The amino-acid sequence in the dimerization domain of the enzyme is the least conserved across species and contributes to the diverse substrate specificity among the various CMAS homologs (Mosimann *et al.*, 2001 ▸; Supplementary Fig. S1).

The active site formed at the dimer interface includes residues from the globular domain of one protomer and the dimerization domain of the opposite protomer. It features a polar nucleotide-binding pocket and a hydrophobic sialic acid-binding pocket. In the absence of CDP and Neu5Ac, apo VcCMAS crystallizes in an ‘open’ conformation (Fig. 2 ▸ *b*) with the catalytic Arg169 residue positioned far away from the active site. In the presence of CDP the dimerization domain of the opposite protomer closes in on the active site, resulting in a ‘partially closed’ conformation (Fig. 2 ▸ *d*). As a consequence, the CDP-bound VcCMAS monomers have a larger dimer interface (1699 Å^2^) compared with the apo structure (1429 Å^2^).

### 3.3. Mononucleotide-binding pocket   

**Figure 3:**
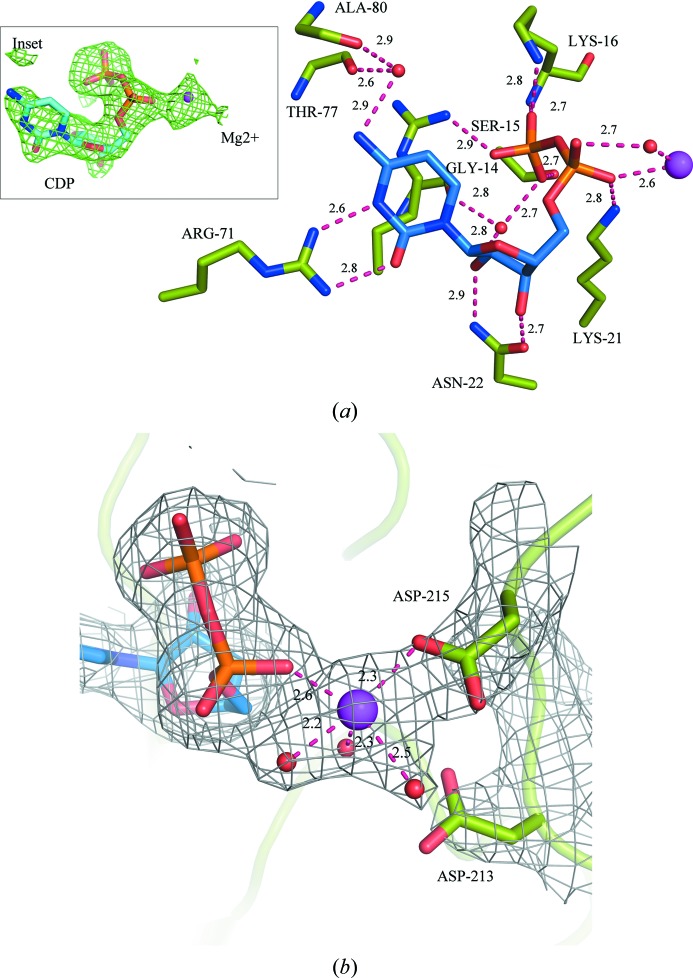
The mononucleotide-binding pocket in VcCMAS. (*a*) The residues that contact the bound CDP are shown in stick representation. Hydrogen bonds are shown as red dashed lines with distances in Å. Mg^2+^ is represented as a magenta sphere. CDP and Mg^2+^ are encased in an *F*_o_ − *F*_c_ electron-density map contoured at 3.0σ (inset). (*b*) Mg^2+^ interacts with the invariant Asp215, the α-phosphoryl group of CDP and three water molecules. CDP, Mg^2+^ and the metal-coordinating residues are encased in a 2*F*_o_ − *F*_c_ electron-density map contoured at 1.0σ.

Similar to the homologous NmCMAS structure (Mosimann *et al.*, 2001 ▸), the mononucleotide-binding site in VcCMAS is comprised of a large cleft formed of residues 11–20 in the P loop following the β1 strand, residues 70–80 in the loop between β5 and helix D, and residues in the C-terminal β11 strand. In the NmCMAS structure (PDB entry 1eyr; Mosimann *et al.*, 2001 ▸), the substrate analog CDP adopts two distinct conformations (I and II) in which the cytosine and the ribose occupy the same positions but the α- and β-phosphoryl groups occupy different positions. However, in the VcCMAS structure CDP binds only in a single conformation that closely mimics conformation I in the structure with PDB code 1eyr (inset in Fig. 3 ▸ *a*). CTP also adopts a similar conformation in the recently released NmCMAS structure (PDB entry 6ckk; M. M. Matthews, H., Yu, Y. Li, X. Chen & A. J. Fisher, unpublished work). The nucleotide-binding residues in subunit *C* and the bound CDP molecule (CDP 1) have well ordered electron density with low temperature factors and thus were used in structural analyses.

Several van der Waals interactions and hydrogen bonds stabilize the nucleotide in the active site. Hydrogen-bond interactions between the conserved Arg71 guanidium N^η1^ and N^η2^ atoms and the N3 and O5 atoms of the cytosine base impart selectivity of the enzyme towards CTP. The N1 atom of the cytosine base interacts with the carbonyl O atoms of Ala80 and Thr77 via a water molecule. The 2′-OH and 3′-OH groups of the ribose moiety of CDP hydrogen-bond to the side-chain carboxamide atoms of the conserved Asn22 and possibly discriminate between dCTP and CTP (Mosimann *et al.*, 2001 ▸). Several conserved residues in the P loop such as Arg12, Gly14, Ser15, Lys16 and Lys21 interact with the α- and β-phosphoryl groups of the nucleotide. The guanidium N^η1^ atom of Arg12 donates a hydrogen bond to the β-phosphoryl O19 atom (Fig. 3 ▸ *a*). The Lys16 main-chain amide group and the Ser15 side-chain hydroxyl group are both at potential hydrogen-bonding distances from the β-phosphoryl O21 atom. The invariant Lys16 ∊-amino group donates a hydrogen bond to the β-phosphoryl O20 atom. In our structure, we observe the side chain of Lys16 on top of the CDP molecule (in subunit *C*) and this possibly protects the nucleotide from solvent exposure and spontaneous hydrolysis. In VcCMAS, the ∊-amino group of Lys21 is at a hydrogen-bonding distance from the α-phosphoryl O16 atom and the β-phosphoryl O20 atom (Fig. 3 ▸ *a*). Our findings strongly support the kinetic data obtained for the homologous *E. coli* CMAS enzyme, in which a K16A mutation resulted in considerable attenuation of the enzyme activity and a K21A mutation completely abolished enzyme activity (Stoughton *et al.*, 1999 ▸). All of the hydrogen-bond interactions between CDP and the VcCMAS enzyme are detailed in Supplementary Table S1.

### 3.4. The site of Mg^2+^ binding and its role in catalysis   

All CMAS enzymes require divalent cations (Mg^2+^ or Mn^2+^) for optimal activity (Horsfall *et al.*, 2010 ▸; Sellmeier *et al.*, 2015 ▸; Mertsalov *et al.*, 2016 ▸). Biochemical and structural data on the closely related lipopolysaccharide-specific (L) and capsule-specific (K) CMP-Kdo synthetases (L-CKS and K-CKS, respectively) from *E. coli* (Heyes *et al.*, 2009 ▸), *H. ducreyi* (Samuels *et al.*, 1999 ▸) and *Aquifex aeolicus* (AA-LCKS; Schmidt *et al.*, 2011 ▸) suggest the involvement of two Mg^2+^ ions in the catalytic reaction. Mg-A activates the sugar hydroxyl group of the substrate 3-deoxy-d-manno-2-octulosonic acid (Kdo) and Mg-B stabilizes the leaving pyrophos­phate group (Schmidt *et al.*, 2011 ▸; Heyes *et al.*, 2009 ▸; Jelakovic *et al.*, 1996 ▸). Additionally, mutational studies on NmCMAS led Horsfall and coworkers to propose that CMAS enzymes also adopt the same catalytic mechanism (Horsfall *et al.*, 2010 ▸). However, Mg^2+^ has not been reported in the active site of either the NmCMAS or the murine CMAS structures.

We observe well defined *F*_o_ − *F*_c_ electron density indicative of a metal ion close to the α-phosphate of CDP and the putative Mg^2+^-binding residues Asp213 and Asp215 (analogous to Asp209 and Asp211 in NmCMAS) in protomer *C*. Along with the Mg^2+^ ion at this site, several water molecules interacting with the phosphoryl group of the nucleotide and the metal ion were also identified. The temperature factor of Mg^2+^ (63 Å^2^) is similar to those of the surrounding residues. In the other protomers, a tightly bound water molecule replaces the Mg^2+^ ion.

In this work, we report the presence of an Mg^2+^ ion, Mg-A, in the metal-binding site of CMAS enzymes. The Mg^2+^ ion coordinates to the side-chain carboxyl O atom (OD1) of the invariant Asp215, the α-phosphoryl O atom of CDP and three water molecules (Fig. 3 ▸ *b*). Based on the observation that a D209A mutation shows a more deleterious effect on the enzyme activity of NmCMAS compared with a D211A mutation, Horsfall and coworkers proposed that Asp209 (which is equivalent to Asp213 in VcCMAS) engages in a bidentate interaction with Mg^2+^, while Asp211 (equivalent to Asp215) participates in a monodentate interaction (Horsfall *et al.*, 2010 ▸). They also suggested that Mg^2+^ does not remain in a fixed position but may occupy different ligation positions during the course of the reaction. In the VcCMAS structure, we observe that Asp215 makes a monodentate interaction with the metal, in contrast to the proposed Asp213, although the latter is seen in the close vicinity and would be equally capable of accepting Mg^2+^ (4.0 Å; Fig. 3 ▸ *b*). Since the Mg^2+^ ion interacts with both the protein and the α-phosphoryl group of CDP and is situated adjacent to the catalytic 2′-OH group of the modeled Neu5Ac, we designate this Mg^2+^ ion as the catalytic ion that is responsible for activating the hydroxyl group of Neu5Ac. Similar to the AA-LCKS structure (Schmidt *et al.*, 2011 ▸), our data also show that the binding of Mg-A is not dependent on Neu5Ac binding. Although Mg^2+^ typically forms an octahedral geometry in complex with protein molecules, we report an incomplete coordination sphere with a coordination number of five. This is not an unusual observation as our structure is at medium resolution and completion of the coordination sphere has been positively correlated with the the resolution limit of the diffraction (Zheng *et al.*, 2008 ▸).

We observe that Mg^2+^ occupies a different position in the VcCMAS structure compared with the CMP-Kdo synthetase (CKS) structures (PDB entries 2y6p and 1gq9; Heyes *et al.*, 2009 ▸; Schmidt *et al.*, 2011 ▸). In the CKS structures the Mg^2+^ ion engages in monodentate interactions with both of the catalytic aspartate residues (Asp95/Asp98 and Asp219/Asp225; Supplementary Fig. S4*a*), whereas in the VcCMAS structure it only interacts with Asp215. Similar to Mg^2+^ in VcCMAS, the Ca^2+^ ion in the recently released NmCMAS structure (PDB entry 6ckk) is seen to interact only with Asp211 (analogous to Asp215 in VcCMAS; Supplementary Fig. S4*b*). Thus, in contrast to the closely related CKS enzymes, in which the metal ion interacts with both of the catalytic aspartate residues, in the CMAS enzymes (PDB entries 6ifd and 6ckk) the metal ion (Mg^2+^/Ca^2+^) interacts primarily with only one aspartate residue (Asp215/Asp211). This could represent one snapshot of the entire catalytic cycle in which the metal ion is seen to engage with only one of the two catalytic aspartate residues. However, there is also a possibility that the mode of metal ion binding differs between CMAS and CKS enzymes.

### 3.5. Conformational changes observed upon CDP binding   

CMAS catalyzes a sequential ordered reaction in which CTP and Mg^2+^ bind prior to Neu5Ac (Samuels *et al.*, 1999 ▸; Horsfall *et al.*, 2010 ▸). The CMAS enzyme shuttles between an ‘open’ and a ‘closed’ conformation to facilitate the entry and the release of substrates from the active site and catalysis, respectively (Mizanur & Pohl, 2008 ▸; Sellmeier *et al.*, 2015 ▸). The major difference between these conformations is the closing in of the dimerization domain onto the active site of the opposite protomer. In murine CMAS, this movement brings the catalytic arginine (Arg202) within hydrogen-bonding distance of the carboxylic acid group of sialic acid and the Asp247 residue of the opposite protomer (Krapp *et al.*, 2003 ▸). Structural evidence from the CDP-bound NmCMAS (PDB entry 1eyr) and CMP-sialic-acid-bound murine CMAS (PDB entry 1qwj) structures suggests that binding of Neu5Ac triggers a conformational change in the dimerization domain of the enzyme leading to active-site closure (Krapp *et al.*, 2003 ▸). As evident from the previously published CDP-bound NmCMAS structure (Mosimann *et al.*, 2001 ▸), nucleotide binding does not initiate this ‘open’ to ‘closed’ movement. A recently released structure of NmCMAS with CTP (PDB entry 6ckk) also captured the enzyme in an ‘open’ conformation.

**Figure 4:**
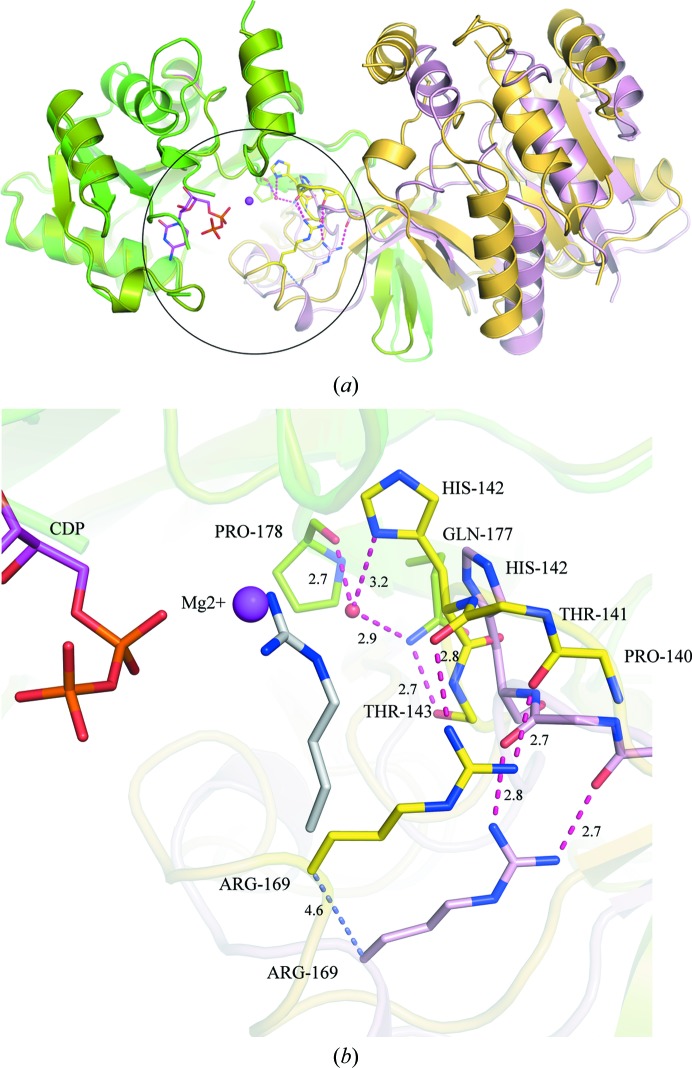
Conformational changes observed upon CDP binding in VcCMAS. (*a*) Superimposition of the apo VcCMAS dimer (protomers colored split pea and pink) on the CDP-bound form (protomers colored gold and yellow) illustrates the movement of the dimerization domain, which places the catalytic Arg169 close to the active site. The circle marks the dimerization domain and the active site. CDP and the Mg^2+^ ion are shown in stick representation and as a sphere, respectively. (*b*) The molecular interactions that stabilize the dimerization domain movement include hydrogen-bond interactions between Gln177 and Thr143 and between Pro178 and His142 via a water molecule. The backbone of the catalytic Arg169 moves 4.6 Å closer (blue dashed line) to the active site and the side-chain guanidino group maintains its interactions with the backbone of Pro140 and Thr141. Superimposition of CMP- and Neu5Ac2en-bound NmCMAS (PDB entry 6ckl) on the VcCMAS structure suggests that the side chain of the catalytic arginine (gray) moves towards the active site only upon substrate binding. Hydrogen bonds are shown as red dashed lines and their lengths are shown in Å.

In contrast, we observe the CDP-bound VcCMAS structure in a ‘partially closed’ conformation (Fig. 4 ▸ *a*). Upon CDP binding, the dimerization domain moves towards the opposite protomer, causing the backbone of the catalytic Arg169 (which is equivalent to Arg202 in murine CMAS and Arg165 in NmCMAS) to shift more than 4 Å closer to the active site. The guanidium group of Arg169 still maintains hydrogen-bond interactions with the backbone carbonyl O atoms of Pro140 and Thr141, as seen in the apoenzyme (Fig. 4 ▸ *b*). The loop that harbors these two residues also moves closer, bringing His142 near the active site. The imidazole N atom of His142 interacts with the carbonyl O atom of Pro178 of the opposite protomer via a water molecule. Also, the side-chain carboxamide N atom of Gln177 interacts with the main-chain carbonyl O atom of Thr143 from the opposite protomer (Fig. 4 ▸ *b*). This interaction is unique to VcCMAS. Gln177 is substituted by an arginine (Arg173) in NmCMAS and a tyrosine (Tyr210) in murine CMAS, and none of them interact with residues from the opposite protomer. The binding of CDP reorganizes water molecules in the active site, which facilitates solvent-mediated interactions at the dimer interface and stabilizes the partially closed conformation of the enzyme.

We also analyzed the packing interactions of the VcCMAS molecules in order to elucidate whether such interactions could have caused domain closure. However, we found it difficult to infer whether packing interactions caused such a movement or whether such interactions merely stabilized this intermediate state. Moreover, the crystal-packing interactions in the NmCMAS crystals (PDB entries 1eyr and 6ckk) are very different from those in the VcCMAS crystals (PDB entry 6ifd). We therefore hypothesize that the partially closed CDP–VcCMAS structure represents an intermediate conformation, fully acknowledging the caveat that this conformation could also have been trapped owing to crystal packing.

In order to identify the dynamics of the domain movement observed upon CDP binding and the residues involved in the hinge-bending motion, we performed *DynDom* analyses (Hayward & Berendsen, 1998 ▸) on the apo VcCMAS (conformer I) and CDP–VcCMAS (conformer II) structures. *DynDom* identified two dynamic domains: the globular domain as the fixed domain (domain 1; residues 1–138 and 179–228) and the extended dimerization domain as a moving domain (domain 2; residues 139–178). Upon CDP binding, the moving domain bends ∼15–20° around the hinge axis, which results in a partially closed conformation. Residues 139–142 and 175–178 are identified as the hinge residues. Upon CMP-sialic acid binding, murine CMAS undergoes a 25° bending motion around the hinge axis that allows additional interfaces to be formed between the C-terminal helices (H6; Krapp *et al.*, 2003 ▸). In the case of CDP binding in VcCMAS, the lesser extent of this bending motion does not allow such an interface to be formed.

### 3.6. A steady-state kinetic study reveals substrate promiscuity in VcCMAS   

**Figure 5:**
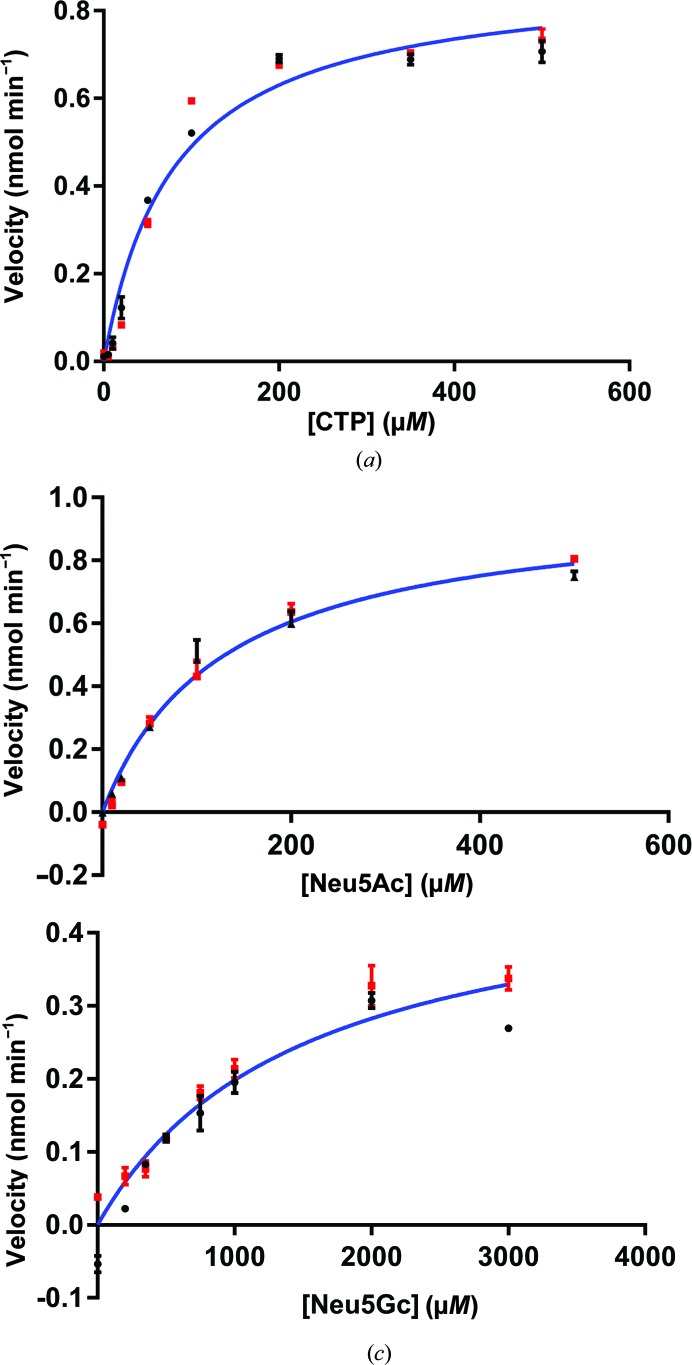
Steady-state kinetic data for the VcCMAS enzyme. *K*_m_ and *V*_max_ determination for CTP (*a*), Neu5Ac (*b*) and Neu5Gc (*c*) with the VcCMAS enzyme was carried out using the EnzCheck pyrophosphatase assay (Webb, 1992 ▸). The data were fitted to the Michaelis–Menten equation using *GraphPad Prism*. The mean and the standard deviation were calculated from two experiments each with duplicate measurements.

We determined the steady-state kinetic constants for CTP, Neu5Ac and Neu5Gc from two independent sets of experiments using nonlinear curve-fitting regression analysis in *GraphPad Prism* (Figs. 5 ▸ *a*, 5 ▸ *b* and 5 ▸ *c*). The kinetic constants obtained for CTP (*K*_m_ = 80 µ*M*, *k*_cat_ = 3.97 s^−1^) are comparable to those for CMAS enzymes from various other bacterial species (Supplementary Table S2). In comparison with Neu5Ac, Neu5Gc is a relatively weaker substrate, with a *K*_m_ that differs by one order of magnitude (127 µ*M* for Neu5Ac versus 1483 µ*M* for Neu5Gc). However, the enzyme has a comparable turnover number for both Neu5Ac and Neu5Gc (*k*_cat_ of 4.46 s^−1^ for Neu5Ac versus 2.21 s^−1^ for Neu5Gc). The specificity constant *k*_cat_/*K*_m_ of 55.75 s^−1^ m*M* ^−1^ for Neu5Ac versus 1.5 s^−1^ m*M* ^−1^ for Neu5Gc reflects the inherent preference of the enzyme for Neu5Ac. Unlike human CMAS, which shows a higher activity for Neu5Gc over Neu5Ac (Lawrence *et al.*, 2001 ▸), the bacterial enzymes vary in substrate specificity and activity. The NmCMAS enzyme has a weaker affinity for Neu5Gc (6 m*M*; Li *et al.*, 2012 ▸) compared with Neu5Ac (0.22 m*M*). Although the NmCMAS and VcCMAS enzymes have comparable turnover numbers for Neu5Ac and Neu5Gc, they show an inherent preference for Neu5Ac over Neu5Gc (Supplementary Table S2). In contrast, the *Clostridium thermocellum* enzyme has comparable *K*_m_, *k*_cat_ and *k*_cat_/*K*_m_ values for both substrates (Mizanur & Pohl, 2008 ▸). In *Streptococcus agalactiae* CMAS, the higher affinity for Neu5Gc over Neu5Ac (16-fold higher) does not reflect the 23-fold lower turnover number for Neu5Gc (Yu *et al.*, 2006 ▸; Supplementary Table S2). To further our understanding of the molecular basis of substrate specificity in VcCMAS, we performed molecular docking with both of the natural substrates.

### 3.7. The structural basis of sialic acid binding and specificity   

The substrates Neu5Ac and Neu5Gc were docked into the active site of the CDP-bound form of VcCMAS using *AutoDock Vina*. We analyzed one of the docked models with the lowest energy. The key interactions between the substrate and the enzyme as observed in the murine CMAS (Krapp *et al.*, 2003 ▸) and NmCMAS (Mosimann *et al.*, 2001 ▸) enzymes are also conserved in VcCMAS.

**Figure 6:**
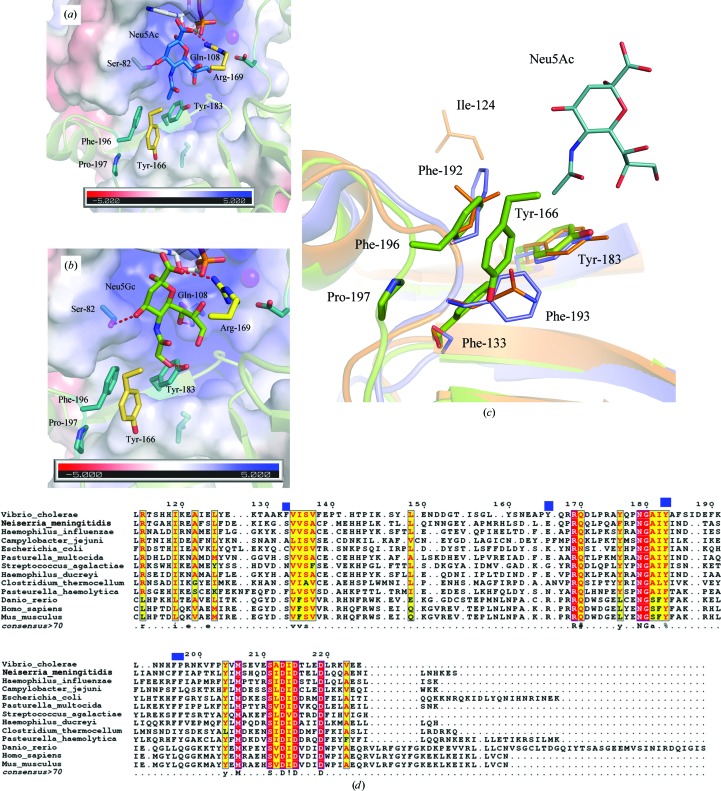
(*a*, *b*) The mode of binding of Neu5Ac and Neu5Gc in the active site of VcCMAS. The electrostatic surface-potential map shows that the hydrophobic residues Phe133, Tyr166, Tyr183, Phe196 and Pro197 constitute the molecular environment around the C5 *N*-acetyl group of Neu5Ac or Neu5Gc. (*c*) A structural comparison of the sialic acid binding pocket between the prokaryotic [NmCMAS (PDB entry 1eyr) and VcCMAS (PDB entry 6ifd)] and eukaryotic (murine CMAS; PDB entry 1qwj) enzymes is shown. Superimposition of all three structures suggests that the loop that harbors the hydrophobic residues (Phe192 and Phe193; NmCMAS nomenclature) differs both in position and in amino-acid sequence. The VcCMAS structure is shown in a cartoon representation in green, NmCMAS in blue and murine CMAS in orange, and residues in the hydrophobic pocket are shown in stick representation. (*d*) A structure-based sequence alignment using *Expresso* (Armougom *et al.*, 2006 ▸) shows much lower conservation of the dimerization domain across bacterial and eukaryotic species. Residues (Phe133, Tyr166, Tyr183, Phe196 and Pro197; VcCMAS nomenclature) located close to the C5 *N*-acetyl moiety of sialic acid are marked with blue rectangles. The CMAS sequences from *N. meningitidis*, *P. multocida*, *P. haemolytica*, *C. thermocellum*, *E. coli*, *H. ducreyi*, *S. agalactiae*, mouse and human were used in this alignment

In the docked model, the functional groups of Neu5Ac and Neu5Gc make several interactions with the conserved residues in the active site. The C1 carboxyl groups of both Neu5Ac and Neu5Gc are at a potential hydrogen-bonding distance from the guanidium group of Arg169. The axial anomeric O2 atom of sialic acid is oriented towards the α-phosphate of CDP; the O4 atom hydrogen-bonds to the Ser82 side-chain hydroxyl group and the O8 atom hydrogen-bonds to the carboxamide N atom of Gln108 (Figs. 6 ▸ *a* and 6 ▸ *b*). Leu106, Tyr166, Tyr183, Phe196 and Pro197 form the hydrophobic pocket that interacts with the C5 *N*-acetyl group of sialic acid (Figs. 6 ▸ *a* and 6 ▸ *b*). These interacting residues vary across the bacterial and eukaryotic enzymes (Fig. 6 ▸ *d*).

Our kinetic data suggest that VcCMAS can catalyze both Neu5Ac and Neu5Gc at a comparable rate and exhibits a tenfold higher affinity for Neu5Ac compared with Neu5Gc. Neu5Gc differs from Neu5Ac in the presence of an additional OH group attached to the C5 *N*-acetyl moiety of Neu5Ac (Fig. 1 ▸). In order to bind Neu5Gc, the enzyme must accommodate the extra OH group in the active site. In VcCMAS, the C5 OH group of Neu5Gc is seen at a potential hydrogen-bonding distance from Tyr183 (equivalent to Tyr179 in NmCMAS; Fig. 6 ▸ *b*). Tyr179/Tyr183 is conserved in the human, mouse and most other bacterial enzymes, apart from those from *Pasteurella haemolytica* and *S. agalactiae* (Fig. 6 ▸ *d*).

In NmCMAS, the bulky hydrophobic residues Tyr179, Phe192 and Phe193 pack against the methyl group of the C5 *N*-acetyl moiety of Neu5Ac and provide specificity for Neu5Ac (PDB entry 6ckl). An F193A mutation in NmCMAS has a deleterious effect on the affinity of the enzyme towards Neu5Ac (a tenfold decrease in *K*_m_; Horsfall *et al.*, 2010 ▸). In fact, Phe192 and Phe193 are conserved in all of the bacterial enzymes with a few exceptions (Fig. 6 ▸ *d*). Although Phe196 is conserved in VcCMAS (analogous to Phe192 in NmCMAS), a smaller proline residue, Pro197, substitutes for the bulky Phe193. Also, the loop between αH and β11, which harbors Phe196 and Pro197, is placed further away from the active site in VcCMAS compared with the NmCMAS and murine CMAS structures. This arrangement provides more space for Neu5Gc in the active site (Fig. 6 ▸ *c*). Findings similar to our docking data are observed in the structure of NmCMAS complexed with CMP and 2-deoxy-2,3-dehydro-*N*-acetyl-neuraminic acid (Neu5Ac2en; PDB entry 6ckl). The murine CMAS enzyme that binds both Neu5Ac and Neu5Gc has a less pronounced hydrophobic pocket compared with NmCMAS, with Tyr216, Leu228 and Gln229 in the active site.

Several differences are observed in the hydrophobic pocket between the NmCMAS and VcCMAS structures. Firstly, the loop harboring Phe192 and Phe193 is placed closer to the sialic acid binding pocket and is stabilized by interactions between the main-chain amide N atoms of the Phe residues (Phe192 and Phe193) and the side-chain carboxyl group of Glu162 of the opposite protomer. In VcCMAS, Tyr166 substitutes for Glu162 and does not engage in any such interaction with the loop. Instead, the Tyr166 side-chain hydroxyl group hydrogen-bonds to Tyr148 of the same protomer. In VcCMAS, the loop harboring Phe196 and Pro197 is kept further away, possibly owing to the presence of the bulky Phe133 at the base. In summary, VcCMAS accommodates Neu5Gc in the active site by creating extra space in the binding pocket.

### 3.8. Binding energetics of ligand interactions   

**Figure 7:**
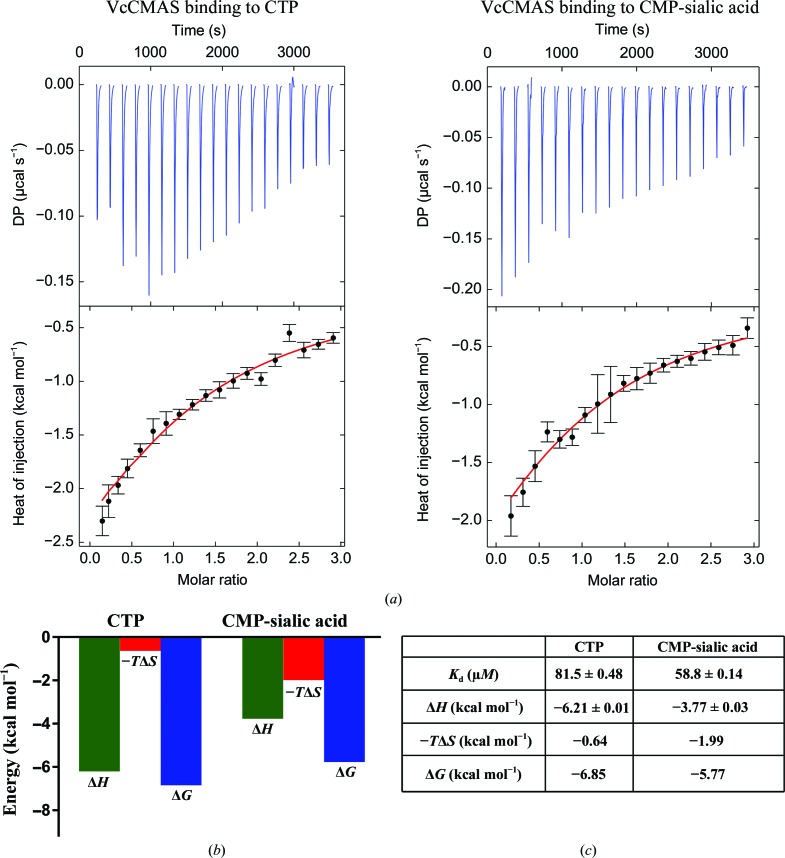
Thermodynamics of the binding of CTP and CMP-sialic acid to the VcCMAS enzyme. (*a*) Titration calorimetry isotherms of VcCMAS with CTP and CMP-Neu5Ac, respectively. The top half of the diagram shows the heat change after each injection versus the molar protein:ligand ratio and the bottom half shows the curve fit in *SEDPHAT*. (*b*, *c*) Thermodynamic signatures of the binding event showing *K*_d_, Δ*H*, −*T*Δ*S* and Δ*G* in graphical and tabular form. All experiments were performed in triplicate (*n* = 3) and the average values are reported.

The thermodynamic ligand-binding parameters provide fundamental information on the protein–ligand interaction event and the intermolecular forces that drive such inter­actions. The molecular mechanism of the binding process can be comprehended by correlating the binding energetics (Δ*G*, Δ*H* and Δ*S*) with the available structural data. Using kinetic and thermodynamic information in conjunction with structural data is a key step in the design and optimization of small-molecule inhibitors. Nucleoside analogs have been instrumental in antiviral and anticancer treatments and also hold great potential as antibiotics (Serpi *et al.*, 2016 ▸). One can envisage the design of targeted inhibitors against CMAS enzymes using CTP and CMP-sialic acid as templates. In order to complement the structural data from crystal structures and docking studies, we carried out ITC experiments (Fig. 7 ▸ *a*) to determine the energetics of ligand binding.

Our ITC data indicate that in the case of CTP binding, enthalpy is a major contributor compared with entropy (Δ*H*_CTP_ = −6.21 kcal mol^−1^, −*T*Δ*S* = −0.64 kcal mol^−1^) in the binding event (Fig. 7 ▸ *c*). The structural data support the thermodynamic data as several hydrogen-bond and van der Waals interactions stabilize CDP in the active site. The dissociation constant (*K*_d_) for CTP, the reciprocal of *K*_a_ or the association constant calculated from the slope of the binding isotherm, is 81.5 µ*M*. Unlike CTP, the binding event of CMP-Neu5Ac has a greater entropic contribution (Δ*H*_CMP-Neu5Ac_ = −3.77 kcal mol^−1^ and −*T*Δ*S* = −1.99 kcal mol^−1^) and the *K*_d_ is 58.8 µ*M* (Fig. 7 ▸ *b* and 7 ▸ *c*). This is not surprising as several hydrophobic interactions stabilize the sialic acid moiety in the active site. The low-micromolar *K*_d_ of the product could explain why the product is the last to leave the active site (Samuels *et al.*, 1999 ▸). As CMAS catalyzes a sequential ordered reaction (Horsfall *et al.*, 2010 ▸), no heat change is observed when only Neu5Ac is titrated against VcCMAS. However, a heat change is observed upon Neu5Ac binding when the enzyme is pre-incubated with CTP (Supplementary Fig. S5).

## 4. Conclusions   

In this study, we have characterized the structural and functional properties of the CMAS enzyme (WP_000064388.1) from *V. cholerae*. Both the apo and nucleotide-bound structures possess all five of the structural motifs responsible for ligand binding in CMAS enzymes. In this work, we have identified the Mg^2+^ ion that interacts with the α-phosphate of CDP and the invariant Asp215 in VcCMAS. Upon CDP binding, the VcCMAS structure shows conformational changes in the dimerization domain, which lead to partial closure of the active site and place the catalytic Arg169 near the Neu5Ac binding pocket. We hypothesize that the partially closed CDP–VcCMAS structure represents an intermediate form, fully recognizing that this conformation could also have resulted owing to crystal packing. However, this intermediate conformation of the enzyme (Supplementary Fig. 3) does support a structural basis for the sequential order of binding of CTP prior to sialic acid in CMAS enzymes.

Our kinetic data reveal that VcCMAS can catalyze both Neu5Ac and Neu5Gc at a comparable rate. In NmCMAS, three bulky hydrophobic residues, Tyr179, Phe192 and Phe193, form the hydrophobic pocket that interacts with the C5 *N*-acetyl group of Neu5Ac. In contrast, the hydrophobic pocket in VcCMAS is less pronounced as the bulky Phe193 is substituted by the smaller Pro197. Additionally, the presence of Phe133 and Tyr166 physically prevents the loop harboring Phe196 and Pro197 from coming forward and constricting the hydrophobic pocket. This arrangement creates extra space in the hydrophobic pocket, which can then accommodate the C5 OH group in Neu5Gc. Moreover, our docking experiment suggests that the polar tyrosyl group of the invariant Tyr183 could potentially hydrogen-bond to the C5 OH group of Neu5Gc.

The overall structural similarity, and in particular that of the nucleotide-binding domain, of VcCMAS to the bacterial and eukaryotic enzymes suggests an evolutionary relationship. Given that CMAS is very specific towards sialic acid, one may think of designing an inhibitor that exploits both the nucleotide- and sugar-binding pockets. CMP-sialic acid can be used as a template as it binds with a low-micromolar affinity. The conservation of the nucleotide-binding domain in CMAS can be exploited to decrease the off-target binding potential of the inhibitor. At the same time, specificity of the inhibitor towards the bacterial enzyme can be achieved by introducing a different functional group at the C5 *N*-acetyl end or the C7 end of the molecule. In this study, we have determined un­liganded and CDP-bound structures of VcCMAS, provided a detailed picture of the metal, nucleotide and substrate-binding pocket, identified the molecular basis of substrate specificity, deciphered the relative contributions of enthalpy and entropy in the binding process and thereby laid the foundation for the design of a structure-based inhibitor.

Sialo-conjugates containing either natural or structurally modified sialic acid are fascinating tools to understand the biological and physiological properties of sialylated structures. Instead of expensive chemical synthesis of sialo-conjugates, chemo-enzymatic synthesis of different sialic acid derivatives by a ‘one-pot two-enzyme’ approach has widely been used (Yu *et al.*, 2004 ▸). In this context, having an active promiscuous CMAS that can activate and generate various CMP-sialic acid derivatives is of great use. The VcCMAS enzyme has inherent substrate promiscuity towards Neu5Ac and Neu5Gc, which can be exploited for this purpose.

## 5. Accession numbers of the proteins that are studied or referred to (NCBI database)   

*Danio rerio* CMAS, NP_001035342.2; *Homo sapiens* CMAS, NP_061156.1; *Mus musculus* CMAS, NP_034038.2; *Streptococcus agalactiae* CMAS, AAD53077.1; *Escherichia coli* CMAS, WP_001259305.1; *Pasteurella multocida* CMAS, WP_005723432.1; *Vibrio cholerae* CMAS, WP_000064388.1; *Campylobacter jejuni* CMAS, WP_002894653.1; *Clostridium thermocellum* CMAS, WP_003512903.1; *Neisseria meningitidis* CMAS, WP_061726245.1; *Haemophilus ducreyi* CMAS, SEW08179.1; *H. influenzae* CMAS, WP_080316015.1; *Pasteurella haemolytica* CMAS, WP_006251815.1.

## 6. Related literature   

The following references are cited in the supporting information for this article: Bravo *et al.* (2001 ▸), Mizanur & Pohl (2007 ▸) and Vann *et al.* (1987 ▸).

## Supplementary Material

PDB reference: CMP-*N*-acetylneuraminate synthetase, 6ifi

PDB reference: complex with CDP and Mg^2+^, 6ifd

Supplementary Figures and Tables. DOI: 10.1107/S2059798319006831/cb5114sup1.pdf

## Figures and Tables

**Table 1:** Data-processing and refinement statistics Values in parentheses are for the highest resolution shell.

	Apo VcCMAS (PDB entry 6ifi)	CDP–VcCMAS (PDB entry 6ifd)
Data processing		
Space group	*P*4_1_	*P*2_1_2_1_2_1_
*a*, *b*, *c* (Å)	75.25, 75.25, 109.59	93.38, 97.72, 98.76
α, β, γ (°)	90, 90, 90	90, 90, 90
Wavelength (Å)	0.98	0.98
Resolution (Å)	47.87–2.80 (2.95–2.80)	55.73–2.30 (2.38–2.30)
*R*_merge_	0.07 (0.86)	0.07 (0.79)
*R*_p.i.m._	0.03 (0.36)	0.03 (0.39)
Completeness (%)	99.9 (99.9)	99.4 (95.3)
〈*I*/σ(*I*)〉	16.7 (2.4)	14.7 (1.6)
CC_1/2_	0.99 (0.88)	0.99 (0.79)
Total No. of reflections	101590	252420
No. of unique reflections	15106	40532
Multiplicity	6.7 (6.7)	6.2 (4.7)
*B* factor from Wilson plot (Å^2^)	78.58	46.16
Refinement statistics		
Resolution (Å)	25.08–2.80	32.55–2.30
No. of reflections	14951 (356)	40516 (811)
No. of reflections, test set	843 (24)	2056 (50)
*R*_work_/*R*_free_ (%)	21.2/24.0 (26.5/29.6)	20.2/24.0 (23.7/20.3)
No. of non-H atoms		
Protein	3288	6867
Ligands	2	112
Water	66	243
R.m.s. deviations		
Bond lengths (Å)	0.015	0.010
Bond angles (°)	1.85	1.14
Average *B* factors (Å^2^)		
Overall	64.35	55.93
Protein	64.51	55.72
Water	54.62	55.72
Ligands	107.51	68.94
Ramachandran plot		
Favored (%)	97.12	98.42
Outliers (%)	0	0
