# Supplementary material for: Structural and functional characterization of CMP-N-acetylneuraminate synthetase from Vibrio cholerae
Source: Acta Crystallogr D Struct Biol. 2019 May 31;75(Pt 6):564–77. doi: 10.1107/S2059798319006831 (PMC6580227; doi:10.1107/S2059798319006831)
Supplement: Supplementary file 1 [file d-75-00564-sup1.pdf]

STRUCTURAL
BIOLOGY

Volume 75 (2019)

Supporting information for article:

Structural and functional characterization of CMP-*N*-acetyl-neuraminate synthetase from *Vibrio cholerae*

Sucharita Bose, Debayan Purkait, Deepthi Joseph, Vinod Nayak and Ramaswamy Subramanian

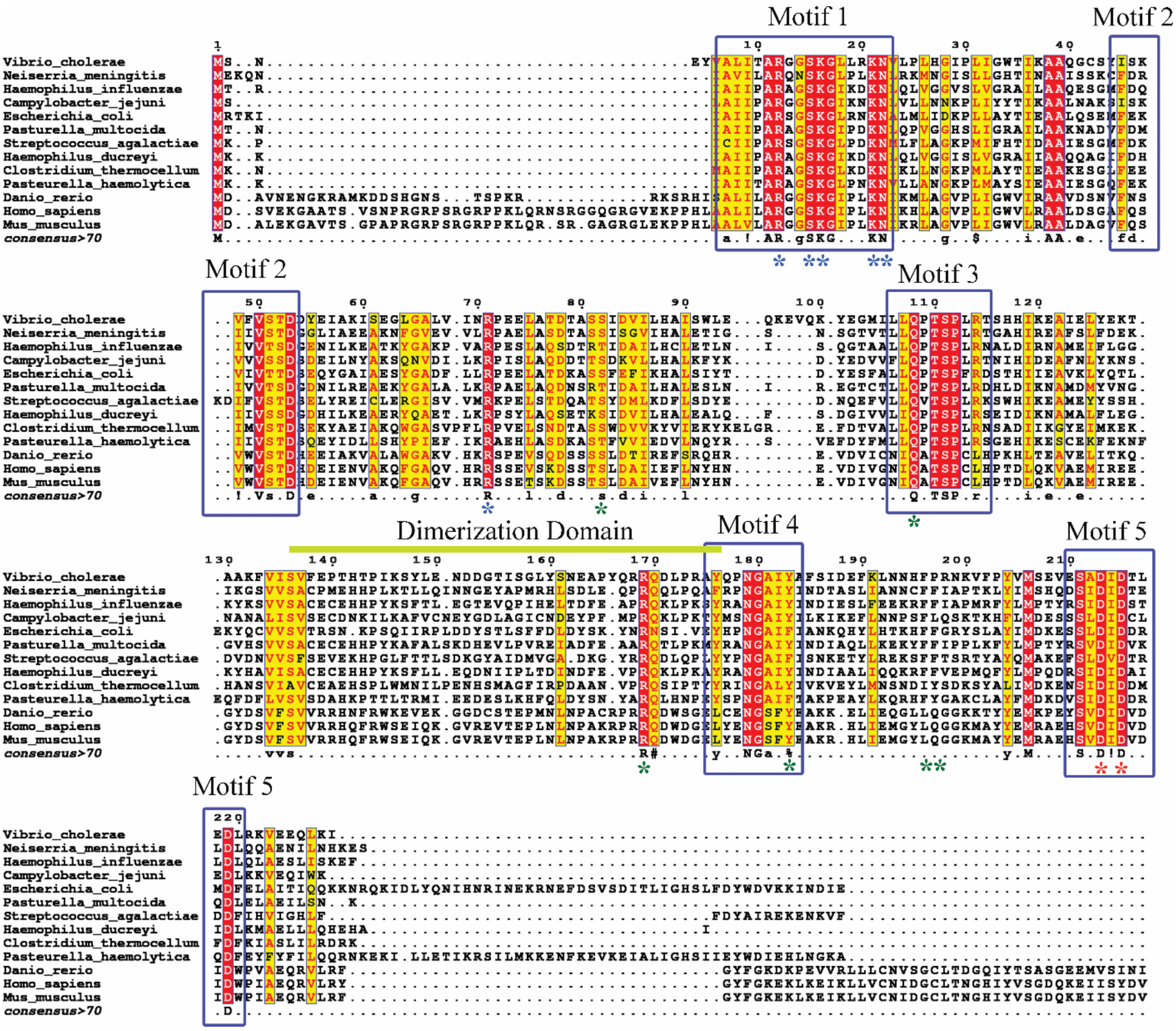

Figure S1 Partial Amino acid sequence alignment of CMAS enzymes from Danio rerio, Homo sapiens, Mus musculus, Streptococcus agalactiae, Escherechia coli, Pasteurella multocida, Vibrio cholerae, Campylobacter jejuni, Clostridium thermocellum, Neisseria meningitidis, Haemophilus ducreyi, Haemophilus influenzae, Pasteurella haemolytica. Sequences were aligned using Tcoffee and displayed by Espript 3. The five hallmark motifs of the CMAS enzymes are labelled (motif 1-5). The fully conserved residues are colored red and semi-conserved residues are colored yellow. Residues that are totally conserved are colored red, semi-conserved residues are colored yellow. Residues interacting with the nucleotide, Neu5Ac and Mg^{2+} are marked with a blue, green and red asterisk respectively. The highly diverse dimerization domain (residues 136-176) is marked with a green line.

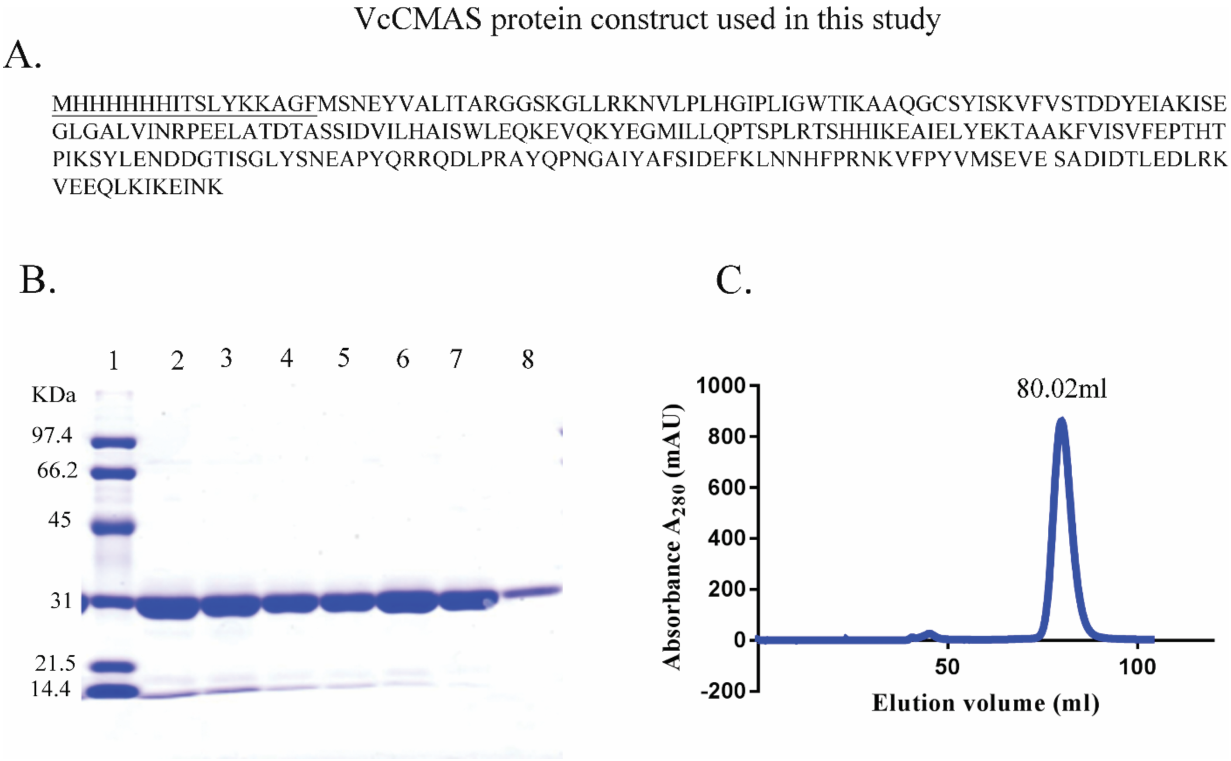

Figure S2 Purification of VcCMAS enzyme. **A.** The amino-acid sequence of the VcCMAS construct used in this study. The N-terminal 6x-His tag and the linker sequence are underlined. **B.** SDS-PAGE data showing various fractions containing the VcCMAS protein. Lane 1. Low range molecular weight marker (Bio-Rad). Lane 2-8. VcCMAS SEC fractions. **C.** Size exclusion chromatography (S200) profile showing that VcCMAS elutes at a volume corresponding to a dimer species.

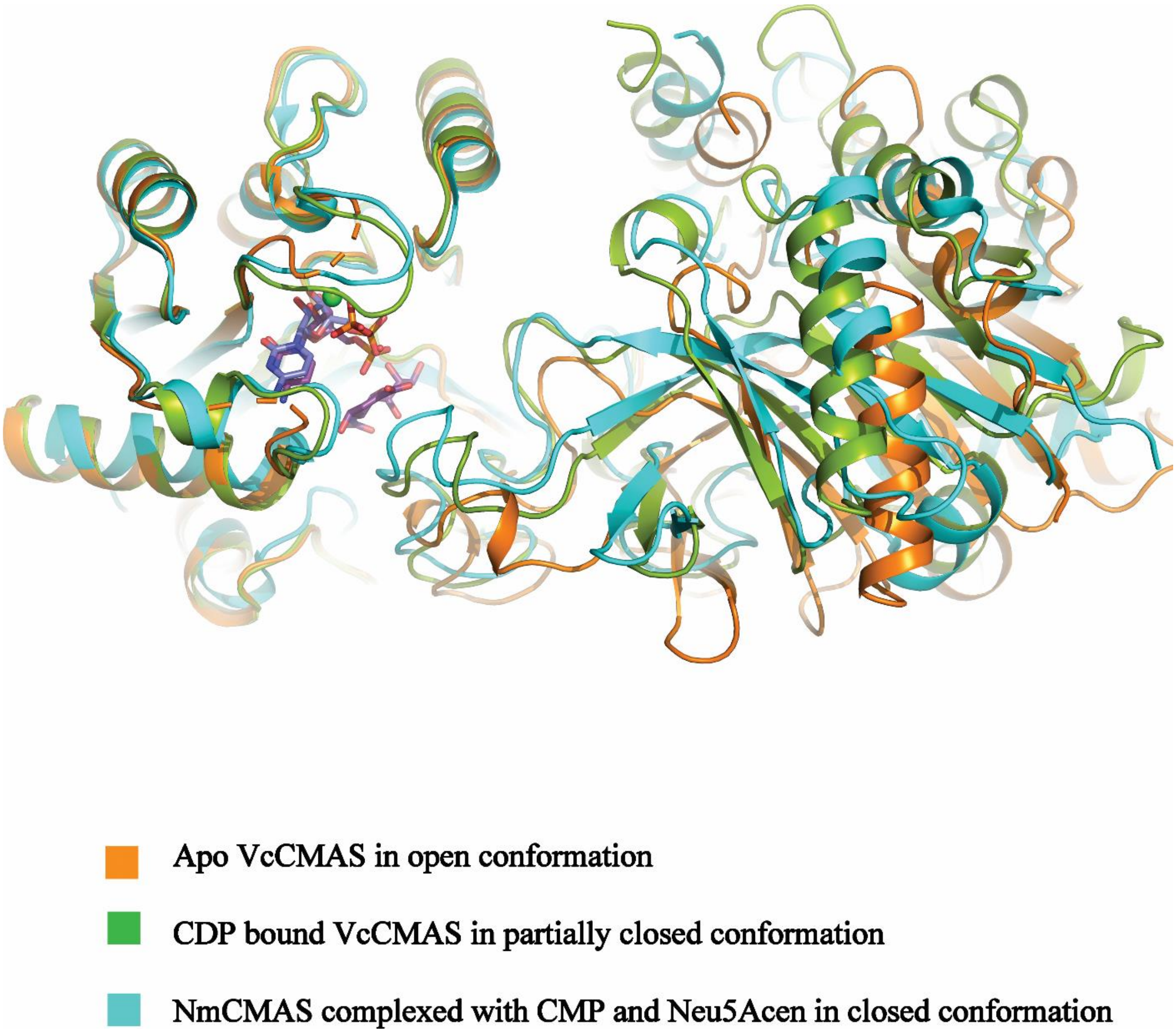

Figure S3 Conformational changes observed in the CMAS dimerization domain in the course of the catalytic reaction. Superimposition of apo and CDP-bound VcCMAS on CMP and Neu5Ac2en-bound NmCMAS (PDB entry 6ckl) shows the following result. Upon CDP binding, the dimerization domain partially closes onto the active site as compared to the apoenzyme. CMP and Neu5Ac2en-bound NmCMAS structure represents the "closed state" of the enzyme, which brings the catalytic arginine close to the sialic acid moiety. CDP, CMP and Neu5Ac2en are represented in blue and magenta sticks respectively, and Mg^{2+} as a green sphere.

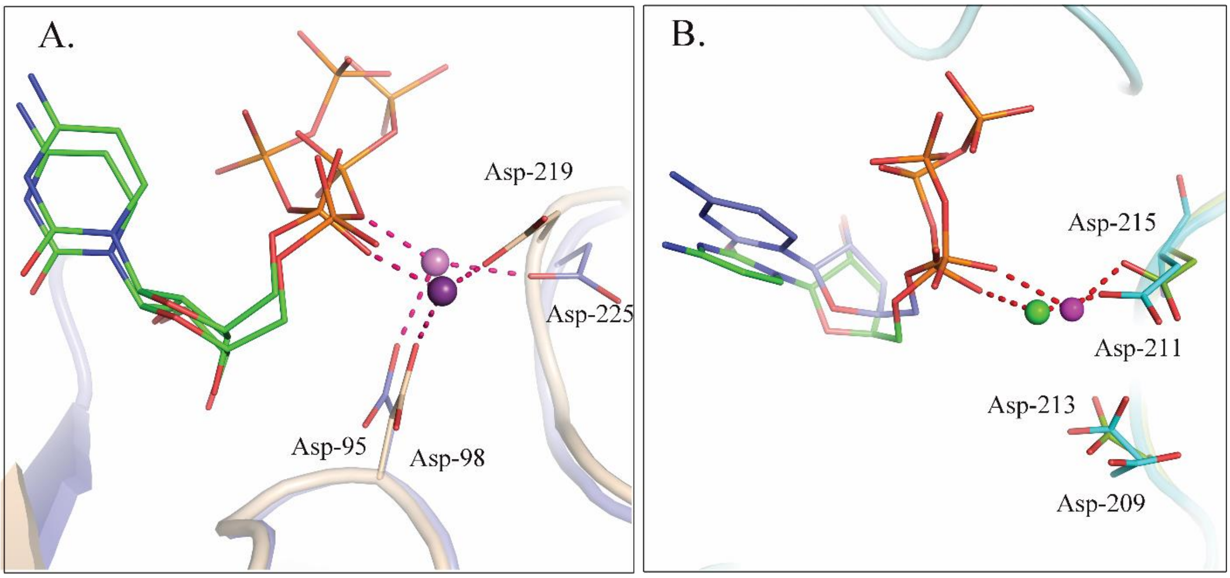

Figure S4 Different mode of metal binding in CKS and CMAS enzymes A. Superimposition of the CTP bound structures of AA-LCKS (2y6p, blue) on *E.coli* LCKS (1gq9,wheat) shows that Mg^{2+} interacts with both the catalytic aspartate residues. In the CTP bound AA-LCKS and *E.coli* CKS structure, Mg^{2+} (pink and violet sphere respectively) engages in monodentate interactions with both the catalytic aspartates (Asp-95 and Asp-219 in AA-LCKS) and (Asp-98 and Asp-225 in *E.coli* LCKS). B Superimposition of CDP-VcCMAS on CTP-NmCMAS (6ckk) structure shows that in both cases, the metal ion interacts with only one of the catalytic Asp residues (Mg^{2+} (magenta sphere) interacts only with Asp-215 in VcCMAS, and Ca^{2+} (green sphere) interacts only the equivalent Asp-211 residue in NmCMAS enzyme. The metal-ligand bonds are represented as red dashes. CDP and CTP molecules are represented as blue and green sticks respectively.

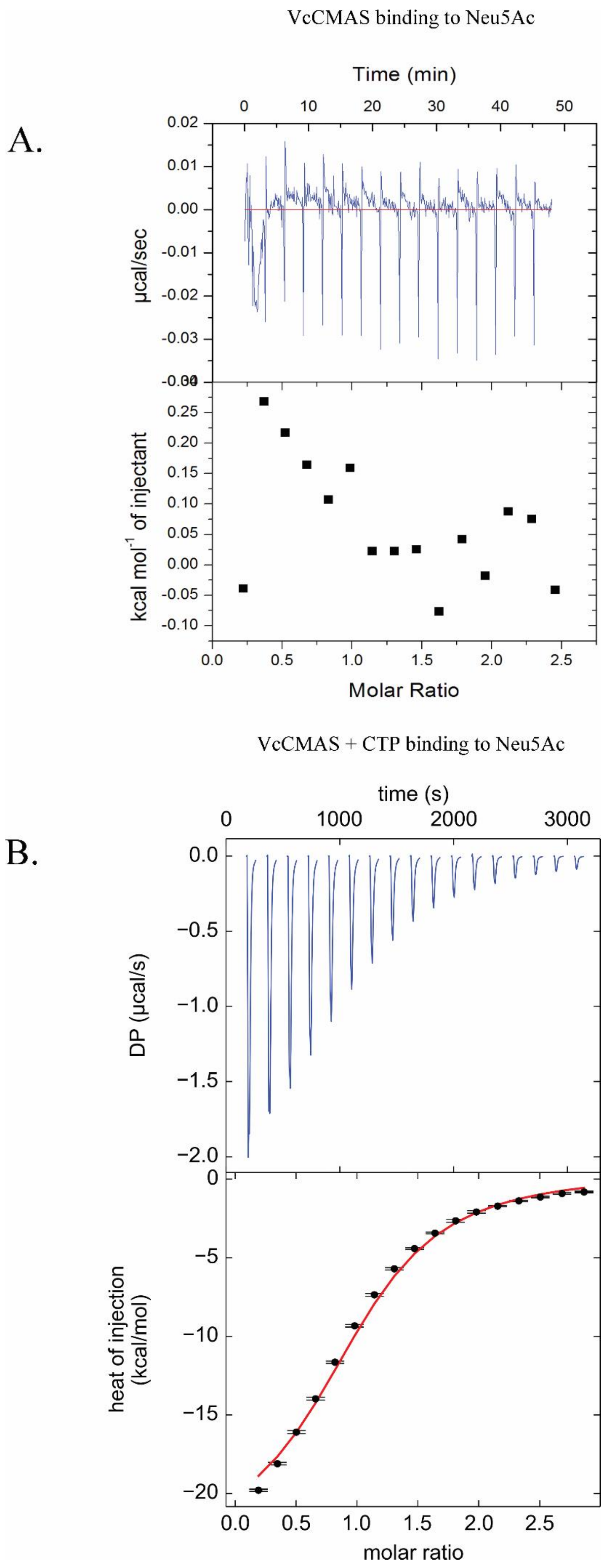
VcCMAS binding to Neu5Ac
A.
Time (min)
µcal/sec
kcal mol-1 of injectant
Molar Ratio
VcCMAS + CTP binding to Neu5Ac
B.
time (s)
DP (µcal/s)
heat of injection (kcal/mol)
molar ratio

Figure S5 Thermodynamics of Neu5Ac binding to the VcCMAS enzyme. **A & B.** Titration Calorimetry Isotherms of Neu5Ac binding to VcCMAS in absence (Figure S5A) and presence of CTP (Figure S5B) respectively. The top half of the diagram shows heat change after each injection vs. the molar ratio of protein: ligand and the bottom half shows the curve fit in Sedphat.

Table S1 Hydrogen bond interactions between CDP and residues in protomer C in VcCMAS are shown.

	**Atoms of CDP**	**Contacting residues in VcCMAS**	**Distance(Å)**
**Cytosine Base**	N1	WAT-116	2.9
	N3	ARG-71 $N^{\eta1}$	2.6
	O5	ARG-71 $N^{\eta2}$	2.8
	O23	ASN-22 N^{δ}	2.9
**Ribose Sugar**	O25	ASN-22 O^{δ}	2.7
		WAT-115	
**α Phosphate**	O15	WAT-227	2.7
	O16	Mg^{2+}	2.6
		LYS-21 N^{ζ}	2.8
		WAT-229	2.7
**β Phosphate**	O19	ARG-12$N^{\eta1}$	2.9
	O20	LYS-16N	2.7
		LYS-16N^{ζ}	2.8
	O21	SER-15O^{γ}	2.7
		WAT-115	2.6
		LYS-21 N^{ζ}	3.1

Table S2 Comparison of the steady state kinetic parameters of VcCMAS with other prokaryotic CMAS enzymes from *Neisseria meningitidis* (Li *et al.*, 2012), *Pasteurella multocida* (Li *et al.*, 2012), *Haemophilus ducreyi* (Li *et al.*, 2012), *Escherichia coli* (Vann *et al.*, 1987), *Pasteurella haemolytica* (Bravo *et al.*, 2001; Mizanur & Pohl, 2007), *Clostridium thermocellum* (Mizanur & Pohl, 2007) and *Streptococcus agalactiae* (Yu *et al.*, 2006). *represents data from this study.

CMAS	K_m(mM)			k_{cat}(s^{-1})			k_{cat}/K_m (s^{-1} $mM^{-1)}$)	
	CTP	Neu5Ac	Neu5Gc	CTP	Neu5Ac	Neu5Gc	Neu5Ac	Neu5Gc
*V. cholerae**	0.08 ±0.01	0.127 ±0.01	1.48 ±0.28	3.97	4.46	2.21	55.75	1.5
N. meningitis	0.59 ±0.1	0.22 ±0.03	6.2 ±1.0	21	19	23	86	3.8
P. multocida	0.24 ±0.03	0.14 ±0.03	n/a	4.6	4.2	n/a	30	n/a
H. ducreyi	0.16 ±0.03	0.18 ±0.03	n/a	5.2	4.8	n/a	27	n/a
E. coli	0.31	4	n/a	n/a	n/a	n/a	n/a	n/a
P. haemolytica	1.77 ±0.16	1.82 ±0.2	3.0 ±0.5	n/a	n/a	n/a	n/a	n/a
C. thermocellum	0.24 ±0.02	0.13 ±0.01	0.16 ±0.01	3.4	2.1	1.6	16.2	10
S. agalactiae	0.96 ±0.05	5.6 ±0.3	0.35 ±0.02	14	16	0.68	2.9	1.9
